# Agricultural Freshwater Pond Supports Diverse and Dynamic Bacterial and Viral Populations

**DOI:** 10.3389/fmicb.2018.00792

**Published:** 2018-04-24

**Authors:** Jessica Chopyk, Sarah Allard, Daniel J. Nasko, Anthony Bui, Emmanuel F. Mongodin, Amy R. Sapkota

**Affiliations:** ^1^Maryland Institute for Applied Environmental Health, University of Maryland School of Public Health, College Park, MD, United States; ^2^Center for Bioinformatics and Computational Biology, University of Maryland, College Park, College Park, MD, United States; ^3^Institute for Genome Sciences and Department of Microbiology and Immunology, University of Maryland School of Medicine, Baltimore, MD, United States

**Keywords:** viral metagenomics, 16S rRNA, bacteriophage, polymerase A, microbiota, virome

## Abstract

Agricultural ponds have a great potential as a means of capture and storage of water for irrigation. However, pond topography (small size, shallow depth) leaves them susceptible to environmental, agricultural, and anthropogenic exposures that may influence microbial dynamics. Therefore, the aim of this project was to characterize the bacterial and viral communities of pond water in the Mid-Atlantic United States with a focus on the late season (October–December), where decreasing temperature and nutrient levels can affect the composition of microbial communities. Ten liters of freshwater from an agricultural pond were sampled monthly, and filtered sequentially through 1 and 0.2 μm filter membranes. Total DNA was then extracted from each filter, and the bacterial communities were characterized using 16S rRNA gene sequencing. The remaining filtrate was chemically concentrated for viruses, DNA-extracted, and shotgun sequenced. Bacterial community profiling showed significant fluctuations over the sampling period, corresponding to changes in the condition of the pond freshwater (e.g., pH, nutrient load). In addition, there were significant differences in the alpha-diversity and core bacterial operational taxonomic units (OTUs) between water fractions filtered through different pore sizes. The viral fraction was dominated by tailed bacteriophage of the order *Caudovirales*, largely those of the *Siphoviridae* family. Moreover, while present, genes involved in virulence/antimicrobial resistance were not enriched within the viral fraction during the study period. Instead, the viral functional profile was dominated by phage associated proteins, as well as those related to nucleotide production. Overall, these data suggest that agricultural pond water harbors a diverse core of bacterial and bacteriophage species whose abundance and composition are influenced by environmental variables characteristic of pond topology and the late season.

## Introduction

Growing urbanization and climate variability have placed immense pressure on the finite supply of groundwater available for agricultural irrigation. As a result, the exploration of alternative irrigation water sources, including recycled water and pond water, has become a global priority (Scheierling et al., 2006; Parsons et al., 2010; Ortega-Reig et al., 2014). While there is no universal standard, ponds are generally defined as small (1 m^2^ to ∼50,000 m^2^), shallow, standing water bodies that can either permanently or temporarily collect freshwater (Biggs et al., 2005; De Meester et al., 2005; Céréghino et al., 2008). These small water bodies are known to house a rich tapestry of aquatic plant and macroinvertebrate species, even greater than that of other larger water bodies (e.g., lakes and rivers) (Biggs et al., 2005). Moreover, the high terrestrial and aquatic interchange of ponds may enable both small free-living (<1 μm) and large/particle-associated bacteria to proliferate (Søndergaard et al., 2005; Roiha et al., 2016). Therefore, assessing the microbial diversity and interactions of these complex water bodies is a critical first step with regard to completing a comprehensive characterization of pond biodiversity, and evaluating the suitability of pond freshwater for agricultural applications, such as the irrigation of food crops.

A growing body of literature has defined several bacterial phyla that are abundant in freshwater and markedly different from that of marine systems (Zwart et al., 2002; Newton et al., 2011). Previous studies have reported a widespread distribution of *Actinobacteria* (lineage acI and acIV) among various freshwater sites, in some cases composing greater than 50% of the total bacterial community abundance (Glöckner et al., 2000; Newton et al., 2011). The *Proteobacteria* phylum in freshwater is also abundant, composed largely of *Betaproteobacteria*. This is in contrast to marine systems where *Alphaproteobacteria* typically dominates (Newton et al., 2011). Furthermore, aquatic microbial communities are influenced by a variety of seasonal factors, such as pH, temperature, and water retention time (Yannarell et al., 2003; Lindström et al., 2005), as well as top-down regulation from predators (e.g., bacteriophage) (Hennes et al., 1995; Chow et al., 2014). However, the majority of studies on the bacterial community composition of freshwater habitats come from large lakes and rivers, and very few have included an analysis of viral populations.

Bacteriophage, viruses that infect bacteria, are the most abundant biological entities in aquatic systems and play an important role in microbial community composition and ecology (Maranger and Bird, 1995; Wilhelm and Matteson, 2008). For instance, phage lysis results in the release of the host’s internal cellular contents (e.g., organic carbon, nitrogen), which then becomes a part of the pool of dissolved organic material (DOM). This phenomenon, known as the viral shunt, increases the level of available DOM for other microbes and is suggested to promote bacterial respiration and growth (Wilhelm and Suttle, 1999; Suttle, 2007; Bonilla-Findji et al., 2008). Several studies have surveyed viral diversity through the use of widely, although not universally, distributed marker genes, such as *polA.* Family A DNA polymerase, *polA*, which encodes the Pol I protein, is the principal polymerase for phage genome replication, and is suggested to be predictive of viral lifestyle based off a single amino acid substitution (Doublie et al., 1998; Schmidt et al., 2014; Wommack et al., 2015). A phenylalanine (wildtype) or tyrosine at amino acid position 762 (relative to *Escherichia coli*) is predictive of virulent phage (i.e., lytic replication), while a leucine substitution at this site seems to be predictive of a temperate lifestyle (i.e., lysogenic replication) (Schmidt et al., 2014). Other studies have surveyed viral communities and diversity through shotgun sampling of genomic DNA from viral concentrates (Wommack et al., 2015).

Similar to most bacterial analyses, the majority of studies of viral metagenomes (viromes) have been created from marine samples, which have provided astounding insights into how phage affect the ecology and biology of their hosts (Suttle, 2007; Rohwer and Thurber, 2009). In addition, there have been several viromes created from large freshwater lakes in the Artic (de Cárcer et al., 2015), the Antarctic (López-Bueno et al., 2009), France (Roux et al., 2012), North America (Djikeng et al., 2009; Green et al., 2015; Mohiuddin and Schellhorn, 2015; Watkins et al., 2016), and Ireland (Skvortsov et al., 2016). While limited in their scope, these studies have provided some of the first data on freshwater phage ecology, demonstrating that, like their hosts, phage diversity is influenced by environmental factors. However, only a few studies have evaluated freshwater viromes from small lakes/ponds and fewer have looked at freshwater viromes in conjunction with a temporal analysis of fine scale host diversity (Fancello et al., 2013; Saxton et al., 2016).

Therefore, we aimed to assess the bacterial and viral components of a temperate agricultural pond in the Mid-Atlantic, United States during the late growing season (October–December), a time when declining temperature and nutrient levels may impact the structure and function of the microbial assemblages. Specifically, we used 16S rRNA gene and shotgun metagenomic sequencing to: (i) survey the bacterial consortium utilizing different filter pore sizes (1 and 0.2 μm); (ii) characterize the diversity and abundance of the bacteriophage within the viral community; and (iii) compare the phylogeny of pond viromes across time using the phylogenetically relevant, and biologically meaningful, Pol I protein.

## Materials and Methods

### Study Site and Sample Collection

Ten-liter water samples were collected in October 2016, November 2016, and December 2016 from a temperate freshwater agricultural pond in central Maryland, United States (maximum depth of ca. 3.35 meters and a surface area of ca. 0.26 ha). A Honda WX10TA (32 GPM) water pump was used to collect water 15–30 cm below the surface into a sterile polypropylene carboy. Samples were kept in the dark at 4°C and processed within 24 h of collection. In addition, a ProDSS digital sampling system (YSI, Yellow Springs, OH, United States) was used to measure, in triplicate: the water temperature (°C), conductivity (SPC uS/cm), pH, dissolved oxygen (%), oxidation/reduction potential (mV), turbidity (FNU), nitrate (mg/L), and chloride (mg/L).

### Sample Preparation

Viral and microbial fractions were separated through peristaltic filtration followed by an iron-based flocculation and resuspension of viral particles. Two 142 mm polycarbonate in-line filter holders (Geotech, CO, United States), one equipped with a 142-mm diameter Whatman 1 μm polycarbonate filter (Sigma-Aldrich, MO, United States) and one with a 142-mm diameter 0.2 μm membrane filter (Pall Gelman Sciences, MI, United States), were attached via sterile 1.6 mm PVC tubing. Each water sample was then filtered sequentially through the 1 μm polycarbonate filter followed by the 0.2 μm membrane filter using a Watson Marlow 323 Series Peristaltic Pump (Watson-Marlow, Falmouth, Cornwall, United Kingdom). No prefiltration steps were conducted prior to the sample processing described above. After filtration, both filters (1 and 0.2 μm) were dissected into four equal quadrants and stored at -80°C until DNA extraction. The iron chloride procedure (John et al., 2011) was then used on the resulting filtrate to concentrate viral particles. Briefly, 1 mL FeCl_3_ solution (4.83 g FeCl_3_ into 100 ml H_2_O) was added to the filtered pond water and incubated in the dark for 1 h. Flocculated viral particles were then filtered onto 142-mm 1 μm polycarbonate filters (Sigma-Aldrich, MO, United States) and stored at 4°C in the dark until resuspension. For viral resuspension, filters were rocked overnight at 4°C in 10 mL of 0.1M EDTA-0.2M MgCl_2_-0.2 M Ascorbate Buffer, described in detail elsewhere (John et al., 2011). To ensure total removal of free DNA contamination, resuspended viral particles were subjected to a DNase I (Sigma-Aldrich, MO, United States) treatment for 2 h and again passed through a 33-mm diameter sterile syringe filter with a 0.2 μm pore size (Millipore Corporation, MA, United States).

### Viral DNA Extraction and Shotgun Sequencing

For the virome analysis, DNA was extracted from 500 μl of the treated viral concentrate using the AllPrep DNA/RNA Mini Kit (Qiagen, CA, United States) per the manufacturer’s instructions and quantified with a HS DNA Qubit fluorescent concentration assay. For each sample, DNA was used in the tagmentation reaction, followed by 13 cycles of PCR amplification using Nextera i7 & i5 index primers & 2× Kapa master mix per the modified Nextera XT protocol. The final libraries were then quantitated by KAPA SYBR FAST qPCR kit and sequenced on the Illumina HiSeq 4000 (Illumina, San Diego, CA, United States).

### Microbial DNA Extraction, 16S rRNA Gene PCR Amplification, and Sequencing

For the 16S rRNA gene analysis, DNA was extracted from each of the four filter quadrants from the 1 and 0.2 μm filters utilizing an enzymatic and mechanical lysis procedure described in detail elsewhere (Chopyk et al., 2017a,b).

The V3-V4 hypervariable region of the 16S rRNA gene was PCR-amplified and sequenced on the Illumina HiSeq (Illumina, San Diego, CA, United States) utilizing a dual-indexing strategy for multiplexed sequencing developed at the Institute for Genome Sciences (Fadrosh et al., 2014), described in detail elsewhere (Chopyk et al., 2017a,b).

### 16S rRNA Gene Data Analysis

16S rRNA gene reads were initially screened for low quality bases and short read lengths (Fadrosh et al., 2014), paired reads were merged using PANDAseq (Masella et al., 2012), de-multiplexed, trimmed of artificial barcodes and primers, and assessed for chimeras using UCHIME in *de novo* mode, as implemented in Quantitative Insights Into Microbial Ecology (QIIME; version 1.9.1-20150604) (Caporaso et al., 2010). Quality-controlled reads were clustered at 97% *de novo* into operational taxonomic units (OTUs) with the SILVA 16S database (Quast et al., 2012) in QIIME (Caporaso et al., 2010). All sequences taxonomically assigned to chloroplasts were removed from further downstream analysis. When appropriate, data was normalized using metagenomeSeq’s cumulative sum scaling to account for uneven sampling depth (Paulson et al., 2013b).

To visualize the relative abundance of bacterial phyla, stacked bar charts were generated using ggplot2 (Wickham, 2009). In addition, bacterial taxa were summarized at the genera level in QIIME (level = 6) and those with a maximum relative abundance greater than 1% in at least one sample were used to build a heatmap via R (ver. 3.3.2) and vegan heatplus (Ploner, 2012).

Significance tests were conducted using a Tukey’s HSD Test between filter size fractions and among sampling dates. Additionally, Pearson correlation coefficients were calculated to identify associations between the water characteristics and the relative abundance of the bacterial phyla/genera.

Alpha diversity was calculated using the R packages: Bioconductor (Huber et al., 2015), metagenomeSeq (Paulson et al., 2013a), vegan (Oksanen et al., 2007), phyloseq (McMurdie and Holmes, 2013), fossil (Vavrek, 2011), biomformat (McMurdie and Paulson, 2016), and ggplot2 (Wickham, 2009) on data rarefied to an even sampling depth (55,307 sequences) and tested for significance using a Tukey’s HSD Test.

Beta diversity was determined through principle coordinates analysis (PCoA) plots of Bray–Curtis, Weighted and Unweighted UniFrac distances calculated using the R package phyloseq and tested for significance with ANOSIM (9,999 permutations) (Beals, 1984; Clarke, 1993; Lozupone et al., 2011; McMurdie and Holmes, 2013).

Core bacterial OTUs for each filter fraction, and the sample as a whole, were defined as OTUs in 100% of samples determined with QIIME’s compute_core_microbiome.py script (Caporaso et al., 2010). Core OTUs were then visualized using ggplot2, and Krona (Wickham, 2009; Ondov et al., 2011).

### Virome Metagenomic Analysis

The paired-end reads were quality trimmed using Trimmomatic (ver. 0.36) (Bolger et al., 2014), merged with FLASh (ver. 1.2.11) (Magoč and Salzberg, 2011), and assembled *de novo* with metaSPAdes (ver. 3.10.1) without read error correction (Nurk et al., 2017). Taxonomic classifications were assigned to contigs by searching predicted peptide open reading frames (ORFs) against the peptide SEED and Phage SEED databases (retrieved 11/17/2017) (Overbeek et al., 2005). Briefly, peptide ORFs were predicted from virome contigs using MetaGene (Noguchi et al., 2006) and were searched against the SEED and Phage SEED databases using protein-protein BLAST (BLASTp ver. 2.6.0+) (*E* value ≤ 1e-3) (Altschul et al., 1990). Taxonomy was assigned to contigs using ORF BLASTp hits to SEED sequences with NCBI taxonomy IDs. A contig is assigned the NCBI taxonomy ID with the maximum sum bit score across all ORF BLASTp hits in the contig. Scripts performing these assignments are available at https://github.com/dnasko/baby_virome.

Functional classifications were assigned to ORFs by searching predicted peptide ORFs against the same peptide SEED database. Peptide ORFs were searched against the SEED databases using BLASTp (*E* value ≤ 1e-3). ORFs were assigned to a SEED subsystem with the maximum sum bit score among all of the ORF’s hits. Only functions associated with viral hits were considered for this analysis.

Counts for taxonomy and functions identified in each virome are based on normalized contig and peptide ORF abundances, respectively. The abundance for each contig was estimated by recruiting all quality controlled reads to the assembled contigs using Bowtie2 (ver. 2.3.3) in very sensitive local mode (Langmead and Salzberg, 2012). Coverage for each contig was calculated using Samtools depth (Li et al., 2009) and a custom parser available at https://github.com/dnasko/baby_virome. ORF abundances are calculated by computing the coverage of the contig within the ORF’s start and stop coordinates. To compare abundance measurements between viromes they are normalized to account for sequencing effort and assembly/recruitment proficiency. Briefly, each contig/ORF’s coverage is divided by the giga base pairs (Gbp) of reads able to recruit back to contigs/ORFs. Taxonomic and functional data were visualized using ggplot2 (Wickham, 2009). Additionally, Pearson correlation coefficients were calculated to identify the associations between the water characteristics and the abundance of the predicted viral taxa.

For the phylogenetic marker gene, predicted ORFs were queried against Pol I UniRef90 (Suzek et al., 2007) clusters using protein-protein BLAST (BLASTp) (Altschul et al., 1990) with an *E* value cutoff ≤ 1e-5. Significant hits were filtered based on length (≥ 100 amino acids) and then confirmed to be Pol I via NCBI’s Conserved Domain BLAST online tool (Marchler-Bauer et al., 2011). The sequences were then aligned with MAFFT using the FFT-NS-i × 1000 algorithm (Katoh et al., 2002). To obtain biologically meaningful data on the Pol I-containing phage, a region of interest (I547 to N923 in *E. coli* IAI39) containing the Phe762 position relative to *E. coli* IAI39 was selected and used to create an unrooted maximum likelihood tree with 100 bootstrap replicates using Geneious 6.0.5 (Kearse et al., 2012) with PhyML (Guindon and Gascuel, 2003). Those with a Phe762 or Tyr762 were defined as generally virulent phage, while those with a Leu762 were defined as generally temperate (Schmidt et al., 2014). Abundances for each Pol I peptide were calculated as described above.

### Data Deposition

16S rRNA gene sequences were deposited in NCBI Sequence Read Archive under the accession numbers SRX3387709-SRX3387732. Viral metagenomic reads were also deposited in NCBI’s Sequence Read Archive under the accession numbers SRS2698857, SRS2698856, and SRS2698858.

## Results

### Water Characteristics

Water properties are described in **Table 1**. Overall, ambient temperature during sampling, water temperature, nitrate and chloride levels, and turbidity decreased during the study period. Conversely, pH, precipitation levels, conductivity (SPC uS/cm), oxidation/reduction potential (mV), and dissolved oxygen (%) were highest in December.

**Table 1:** Agricultural pond water characteristics during sampling period.

Water property	October	November	December
Ambient temp. (C)	17.2	12.2	3.9
Water temp. (C)	19.8	10.9	7.4
PH	7.7	7.56	8.08
Dissolved oxygen (%)	116.4	96.4	117.7
Nitrate (mg/L)	0.63	0.26	0.19
Chloride (mg/L)	13.8	13.3	7.9
Turbidity (FNU)	30.2	9.6	3.4
Precipitation^†^ (in.)	0	0	0.2
Conductivity (SPC uS/cm)	158.9	160.8	167.1
Oxidation/reduction (mV)	189.7	159.8	243.9

*†24 h prior to sampling.*

### 16S rRNA Gene Sequencing Effort

In total, 24 samples were PCR-amplified for the 16S rRNA gene and sequenced: four quadrants each from the 1 and 0.2 μm filters from each sampling date (October, November, and December). After sequence quality filtering, 7,489 OTUs (97% identity) were identified from a total of 2.5 million sequences across all samples, with an average number of ca. 103,000 (± ca. 36,000 SD) sequences per sample and an average of ca. 2,100 (± ca. 500) OTUs.

### Bacterial Community Composition and Temporal Variations

**FIGURE 1:**
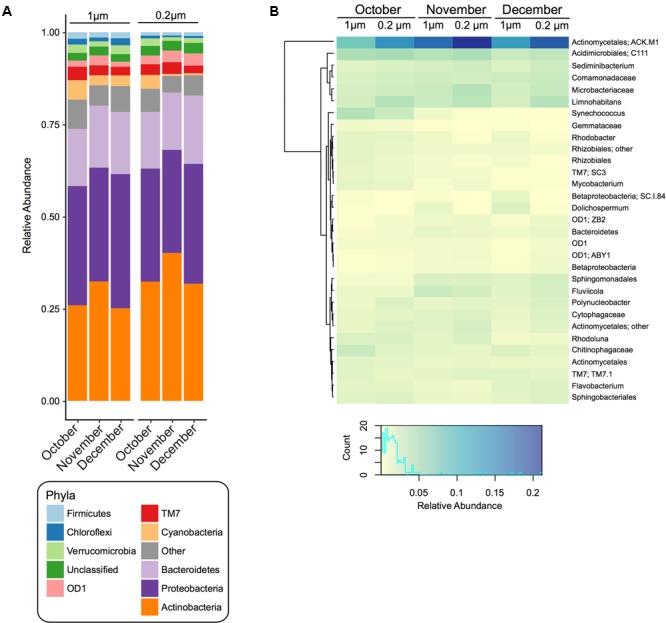
Bacterial community composition and diversity of 1 and 0.2 μm filter fractions over time. **(A)** Stacked bar chart of the relative abundance of the bacterial community composition at the phylum level within pond samples from each month (October, November, and December) and each filter fraction. **(B)** Heatmap based on the relative abundance of the bacterial community composition at the genus level. Displayed are genera or lowest available taxonomic assignments representing more than 1% in at least one of the pond samples. Pooled samples are clustered using Manhattan distance. Color key depicts the spectrum of relative abundance with a histogram of the counts of individual values.

The most abundant pond water phyla were *Actinobacteria, Proteobacteria*, and *Bacteroidetes* in all samples, however, their average relative abundance fluctuated over the time course and between filter pore sizes (**Figure 1A** and Supplementary Table S1, S2). For instance, *Actinobacteria* was significantly (*p* < 0.05) higher at all time points in the 0.2 μm fraction, whereas *Chloroflexi, Firmicutes, Cyanobacteria*, and *Proteobacteria* were significantly higher in the 1 μm fraction at all time points (**Figure 1A** and Supplementary Table S1). From October to December in both fractions the relative abundance of *Bacteroidetes* increased significantly (*p* < 0.05), whereas *TM7* and *Cyanobacteria* decreased (Supplementary Table S2).

At the genera level, summarized via QIIME, there were 31 taxa that were greater than 1% in at least one sample (**Figure 1B**). Of these, 8 had a significantly (*p* < 0.05) higher relative abundance at all time points in the 0.2 μm fraction than the 1 μm fraction (*ACK.M1, Limnohabitans, Microbacteriaceae, Sediminibacterium, Polynucleobacter, Actinomycetales, Cytophagaceae, ZB2*), while six were significantly (*p* < 0.05) greater in the 1 μm fraction compared to the 0.2 μm fraction (*Synechococcus, Chitinophagaceae, Dolichospermum, Rhizobiales, SC.I.84, Gemmataceae*) (**Figure 1B** and Supplementary Table S3).

Moreover, within each fraction there were significant (*p* < 0.05) changes in the relative abundance of the bacterial taxa over the sampling period (**Figure 1B** and Supplementary Table S4). For instance, in the 1 and 0.2 μm fractions, *ACK.M1, Fluviicola, Sphingomonadales, Dolichospermum, Flavobacterium, Bacteroidetes, SC.I.84*, and *Betaproteobacteria* increased significantly (*p* < 0.05) from October to December, while *C111, Synechococcus, Chitinophagaceae, Rhodoluna, Actinomycetales, Rhodobacter, Rhizobiales, Mycobacterium, Rhizobiales, SC3*, and *Gemmataceae* decreased significantly (*p* < 0.05). In addition to the taxa above, in the 1 μm fraction there was also a significant (*p* < 0.05) increase in *Comamonadaceae* and *Polynucleobacter* and a significant decrease in *Sphingobacteriales* and *OD1* between the October and December sampling dates. For the 0.2 μm fraction, there was also a significant (*p* < 0.05) increase in the relative abundance of *Sediminibacterium, Cytophagaceae, ZB2*, and *ABY1* between the October and December sampling dates.

### Relationships Between Water Characteristics and Bacterial Abundance

Despite the small sample size (*n* = 3), several bacterial phyla showed significant correlations (*p* < 0.05) with the measured water characteristics (Supplementary Figure S1A). In both filter fractions, the relative abundance of *Actinobacteria* was negatively correlated with the level of dissolved oxygen. In just the 1 μm fraction, the relative abundance of *Verrucomicrobia* and *Proteobacteria* were positively correlated with dissolved oxygen and pH, respectively, while *TM7* was positively correlated with both water temperature and turbidity. In addition, in just the 0.2 μm fraction, the relative abundance of *Firmicutes* showed positive correlations with pH and oxidation/reduction. Conversely, the relative abundance of *Bacteroidetes, TM7*, and *OD1* were negatively related to, chloride, pH, and nitrate, respectively (*p* < 0.05). At the genera level in both filter fractions, *Synechococcus* was positively correlated with nitrate (Supplementary Figure S1B).

### Bacterial Alpha Diversity

**FIGURE 2:**
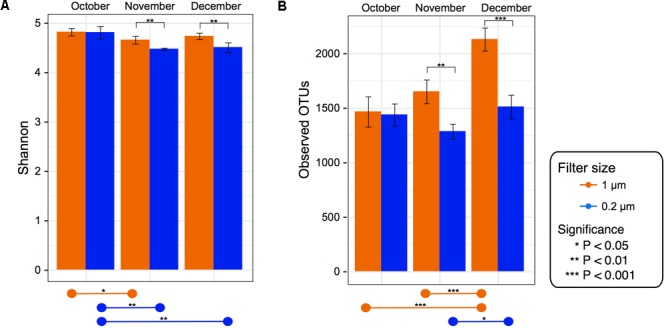
Alpha diversity for each filter fraction in late season pond water. Bar charts of alpha diversity measured using **(A)** Shannon index and **(B)** Observed OTUs. Color denotes filter pore size, either 1 μm (orange) or 0.2 μm (blue). Pairwise significance between filter size denoted by brackets within graph for each date. Significance within each filter among time points denoted by lines at bottom of the figure. Error bars denote standard deviation.

Alpha diversity was computed for Observed OTUs and Shannon diversity and tested for significance between filter size and over time within each fraction (**Figure 2**).

Despite the 0.2 μm fraction containing significantly higher levels of some of the dominant genera, the 1 μm fraction had a significantly higher Shannon index and observed OTU value in the November and December (*p* < 0.05) samples (**Figure 2**). When testing within each fraction over time, the Shannon index values in the 1 μm fraction were significantly (*p* < 0.05) higher in October than November. In the 0.2 μm fraction the Shannon index values were significantly higher in October than both November and December (**Figure 2**). However, the Observed OTU values in the 1 μm fraction were significantly higher in December than both October and November, while in the 0.2 μm fraction December was only significantly higher than November.

### Bacterial Beta Diversity

**FIGURE 3:**
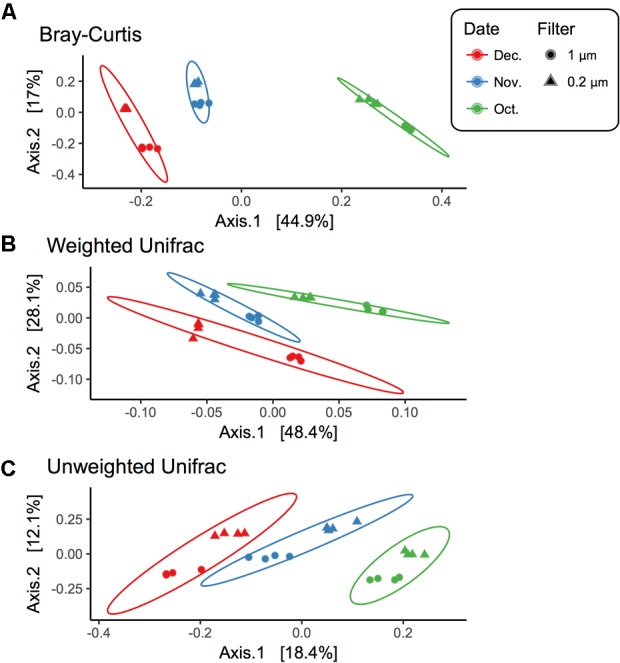
Beta diversity for each filter fraction in late season pond water. PCoA plots of beta diversity measured using **(A)** Bray–Curtis, **(B)** Weighted UniFrac, and **(C)** Unweighted UniFrac. Shape denotes filter pore size, either 1 μm (circle) or 0.2 μm (triangle), and color denotes month when water was sampled, October (green), November (blue), and December (red). Ellipses are drawn at 95% confidence intervals for each month.

Beta diversity comparisons using PCoA plots of Bray–Curtis distances computed for all samples revealed significant clustering by date (*R* = 0.94, *p* = 0.001) along axis 1, which accounted for nearly 45% of the variation (**Figure 3**). Samples along axis 2 (17% of the variation) appeared to cluster by filter size (1 μm vs. 0.2 μm). This trend was also observed both utilizing weighted (*R* = 0.7458, *p* = 0.001) and unweighted (*R* = 0.8524, *p* = 0.001) UniFrac (**Figure 3**) distances. Additionally, within each date, samples clustered by filter pore size: December (*R* = 1, *p* = 0.022), November (*R* = 1, *p* = 0.022), and October (*R* = 1, *p* = 0.029) (Supplementary Figure S2).

### Core OTUs

**FIGURE 4:**
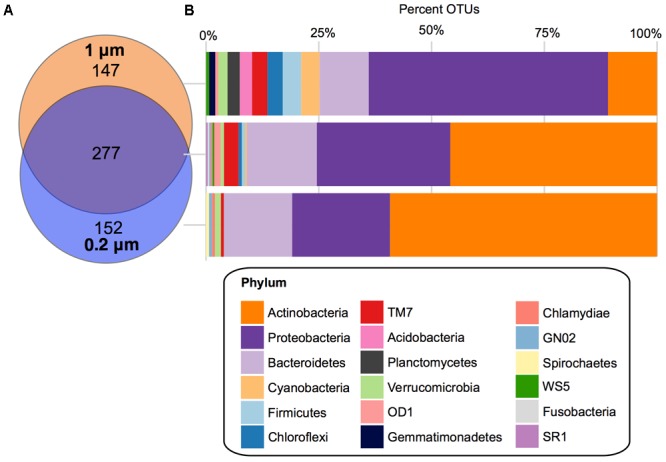
Core OTUs for 1 and 0.2 μm filter fractions. **(A)** Venn diagram depicting the unique and shared OTUs that occurred in 100% of samples in the 1 μm (orange) and 0.2 μm (blue) filter fractions. **(B)** Stacked bar charts of the percentage of OTUs assigned each phylum within the shared and unique cores.

There were 277 core OTUs present in the farm pond water over the 3 months across both fractions (**Figure 4**). These were largely *Actinobacteria* (127 OTUs), followed by: *Proteobacteria* (82 OTUs), *Bacteroidetes* (43 OTUs), TM7 (9 OTUs), OD1 (4 OTUs), *Verrucomicrobia* (2 OTUs), *Firmicutes* (2 OTUs), *Chloroflexi* (2 OTUs), *Cyanobacteria* (1 OTU), *WS5* (1 OTU), *SR1* (1 OTU), *GN02* (1 OTU), *Fusobacteria* (1 OTU), and *Chlamydiae* (1 OTU). For the 0.2 μm fraction, there was a unique core of 152 OTUs, largely *Actinobacteria* (90 OTUs), followed by *Proteobacteria* (33 OTUs), and *Bacteroidetes* (23 OTUs), *Verrucomicrobia* (2 OTUs), *TM7* (1 OTU), *GN02* (1 OTU), *Chlamydiae* (1 OTU), and *Spirochaetes* (1 OTU). The unique core for the 1 μm fraction was more diverse in bacterial phyla compared to the 0.2 μm. It consisted largely of *Proteobacteria* (78 OTUs), followed by *Actinobacteria* (16 OTUs), *Bacteroidetes* (16 OTUs), *Cyanobacteria* (6 OTUs), *Firmicutes* (6 OTUs), *Chloroflexi* (5 OTUs), *TM7* (5 OTUs), *Planctomycetes* (4 OTUs), *Acidobacteria* (4 OTUs), *Verrucomicrobia* (3 OTUs), *Gemmatimonadetes* (2 OTUs), *OD1* (1 OTU), and *WS5* (1 OTU).

When looking at the core bacterial taxa at a lower taxonomic level, in the 0.2 μm fraction the *Actinobacteria* OTUs were dominated by *Actinomycetales* ACK-M1 (70%), followed by *Rhodoluna* (4%) under the family *Microbacteriaceae* (Supplementary Figure S3B). The *Proteobacteria* were largely *Limnohabitans* (24%) and *Polynucleobacter* (30%) and the *Bacteroidetes* were *Sediminibacterium* (22%) and a large unclassified family of *Cytophagaceae* (35%) (Supplementary Figure S3B). Within the unique core of the 1 μm fraction, the only prominent genus of the *Proteobacteria* was *Rhobacter* at 5%, followed by *Novosphingobium* at 3% and 17 other genera (each at 1%) (Supplementary Figure S3A). At a higher taxonomic level, a family of largely unclassified *Rhizobiales* was also abundant (23%). The *Actinobacteria* were dominated by *Acidimicrobiales* C111 (25%), *Solirubrobacterales* (13%), and a large proportion of mostly unclassified *Actinomycetales* (44%). Finally, in the *Bacteroidetes* phylum of the 1 μm fraction there was mostly *Fluviicola* at 13%, as well as *Leadbetterella, Runella*, and *Sediminibacterium*, which were each at 6%.

### Shotgun Sequencing Effort and Assembly

Each sample from the viral fraction was sequenced on the Illumina HiSeq for a total of 89,645,509 read pairs (35,522,822 from October; 32,988,451 from November; 21,134,236 from December). We assembled the reads from all sampling dates to construct a total of 872,200 contigs (272,687 from October; 409,758 from November; 189,755 from December). The mean contig length was 593 nucleotides in October (range from 55 to 366,802 nucleotides), 641 in November (range from 55 to 286,691 nucleotides), and 655 in December (range from 55 to 331,733 nucleotides). The GC content was similar among the three sampling dates: October (Mean 46.51%, Median 45.42%); November (Mean 46.18%, Median 45.59%); and December (Mean 46.12%, Median 45.21%).

### Viral Taxonomic Composition and Abundance

**FIGURE 5:**
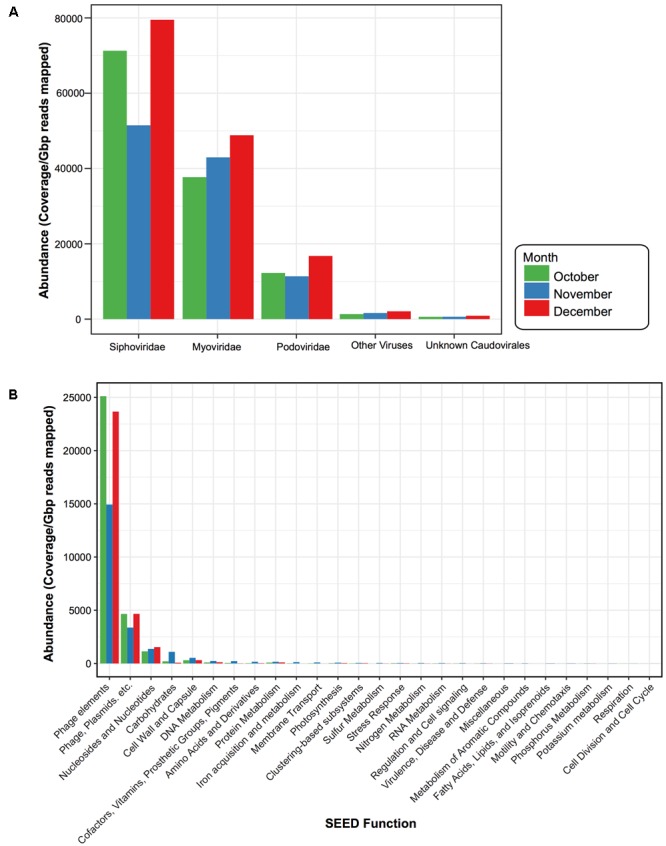
Viral taxonomy and function for each sampling date. Bar plots comparing the normalized abundance of viruses with homology to known **(A)** viral taxa and **(B)** functional SEED categories. Bar color denotes month when water was sampled, October (green), November (blue), and December (red). Phage elements refer to the SEED functional category “Phages, Prophages, Transposable elements” and Phage, Plasmids, etc. denote the SEED functional category “Phages, Prophages, Transposable elements, Plasmids.” Abundance determined by calculating coverage/Gbp reads mapped.

Similar to other studies, a large portion of the assembled contigs within the viral fraction had no known homologs (42% October, 51% November, and 42% December could be assigned taxonomy) (Krishnamurthy and Wang, 2017). For those that did have a hit we calculated the normalized abundance. From this we found that 47% (October), 27% (November), and 53% (December) of the assigned taxonomic composition were homologous to viruses; the vast majority of which were from the dsDNA bacteriophage *Caudovirales* (99%, 98%, and 99%) (**Figure 5A**). Within the *Caudovirales, Siphoviridae* dominated at all time points followed by *Myoviridae* and then *Podoviridae*. Other viral categories included those homologous to ssDNA viruses *Inoviridae*, dsDNA viruses *Tectiviridae, Ligamenvirales, Bicaudaviridae*, and other unclassified bacteriophage (**Figure 5A**). For the dominant viral families (*Podoviridae, Siphoviridae*, and *Myoviridae*), there were no water characteristic that significantly correlated with their abundances; however, there were several bacterial taxa whose relative abundance correlated with the abundance of the dominant viral families (Supplementary Figure S4). For instance, at the phylum level the relative abundance of *OD1* in the 1 μm fraction negatively correlated with the abundance of *Siphoviridae*, while in the 0.2 μm the relative abundance of *TM7* negatively correlated with the abundance of *Podoviridae* (Supplementary Figure S4).

### Viral Functional Composition

ORFs from the virus-assigned contigs were functionally annotated using the SEED Subsystems (Overbeek et al., 2005). Again, to compare these viral categories across the 3 months, we calculated the normalized abundance for each of the ORFs assigned to the SEED functional categories (**Figure 5B**). While ORFs homologous to virulent genes were present (e.g., Multidrug Resistance Efflux Pumps, Zinc Resistance, Copper Homeostasis) they were not abundant within the time period. The majority (93% October, 80% November, and 92% December) were Phage Elements, defined by the SEED subsystem hierarchy (Overbeek et al., 2005) as either “Phages, Prophages, Transposable elements,” which were largely phage structural genes (e.g., capsid, scaffolding) or “Phages, Prophages, Transposable elements, and Plasmids,” which were genes related to phage replication and packaging (e.g., terminase, integrase, helicase, primase). This was followed by “Nucleosides & Nucleotides” (4% October, 6% November, and 5% December), which were largely genes involved in ribonucleotide reduction.

### Viral Marker Gene: Polymerase A

**FIGURE 6:**
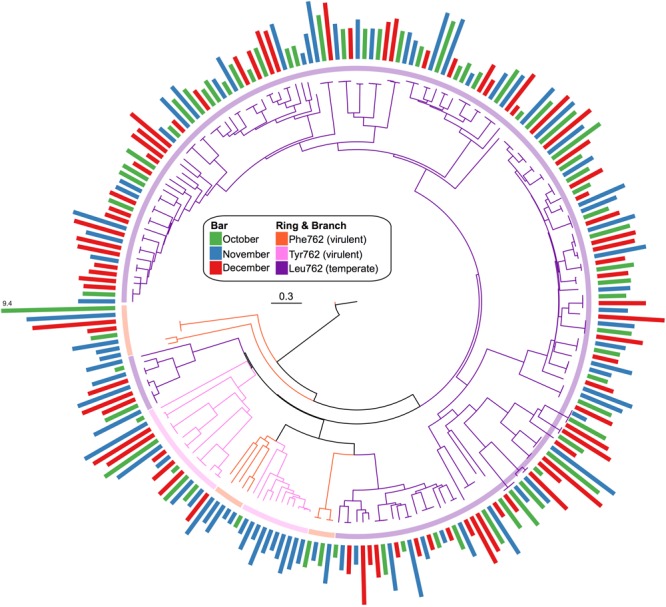
Phylogenic tree of viral Pol I peptides for each sampling date. Unrooted maximum likelihood tree with 100 bootstrap replicates of representative Pol I peptide sequences. Branches and inner ring colored by 762 position residues, Phe762 (orange), Tyr762 (pink), and Leu762 (purple). Radial bar chart represents the log_2_-normalized abundance of each peptide (coverage/Gbp reads mapped) and is colored by sampling date, October (green), November (blue), and December (red).

A total of 842 confirmed Pol I peptides were extracted from our assembled pond water viromes (271 October, 320 November, and 251 December). From these, only 228 spanned the region of interest and were included in the phylogenic analysis: 68 in October, 83 in November, and 77 in December. The phylogenetic analysis (**Figure 6**) showed that the Pol I peptides grouped largely by their 762 position, whereas the sampling dates were distributed among the different clades. Again, to compare the Pol Is we calculated the normalized abundance for each. While the majority of Pol I peptides had the Leu substitution at the 762 site (184), followed by Tyr (27) and then the wildtype Phe (17), the clade at the highest abundance was those with the wildtype Phe mutation. Because this clade was so abundant, we used BLASTp to assess the top hit, which were uncultured bacteriophage from the Dry Tortugas surface water (Schmidt et al., 2014) and the seawater collected from the deep chlorophyll maximum of the Mediterranean Sea (Mizuno et al., 2013) (*E* value < 1e-300).

## Discussion

While ponds represent a potential source of irrigation water, provided adequate filtering and monitoring technologies are employed, it is important to keep in mind their role in the ecosphere. Their small size and shallow depth enables a complex community of aquatic plants and macroinvertebrate species, as well as an interacting community of microorganisms.

However, changing water levels and anthropogenic factors associated with irrigation systems may interrupt the delicate balance of microbial life, which can ultimately impact higher trophic levels. Therefore, it is critical to carefully manage our use, or contamination, of these systems in search of irrigable water.

Here, the studied pond was dominated by *Actinobacteria, Proteobacteria*, and *Bacteroidetes*, common phyla found in most freshwater lakes (Newton et al., 2011). However, as the late season progressed there appeared to be changes in the bacterial community composition that correlated with specific environmental factors. For instance, *Actinobacteria* fluctuated throughout the sampling period, and was negatively correlated with the percentage of dissolved oxygen (**Figure 1A** and Supplementary Figure S1A). A previous study of the Luhuitou fringing coral reef also reported a negative correlation between dissolved oxygen and the abundance of several *Actinobacteria* species that may be due to their anaerobic capabilities (Kuang et al., 2015). This relationship may help to explain the fluctuations in freshwater *Actinobacteria* abundances and diversity described in other seasons (Allgaier and Grossart, 2006). At a lower taxonomic level, the relative abundance of *Synechococcus* decreased significantly throughout the late season (**Figure 1B** and Supplementary Table S4). This is not surprising as the growth rate of *Synechococcus* is known to decrease with temperature and nitrogen levels, which occurred during the sampling period (Sakamoto and Bryant, 1997, 1999).

While the changes in relative abundance described above were apparent in both filter fractions (1 and 2 μm), there were differences in alpha diversity, beta diversity, and core bacterial composition between the two fractions (**Figures 1–3** and Supplementary Figure S1). For instance, the 0.2 μm core microbiota was dominated by *Actinobacteria*, largely *Actinomycetales* ACK-M1, a known representative in most freshwater habitats (Van der Gucht et al., 2005) and free-living *Proteobacteria, Limnohabitans*, and *Polynucleobacter*, (Hahn et al., 2005, 2010; Šimek et al., 2010) (**Figure 4** and Supplementary Figure S3). Whereas, the core of the 1 μm filter fraction was dominated by a diverse set of *Proteobacteria*, largely of the order *Rhizobiales* and *Rhodobacteriales*, previously found to dominate the particle-associated fraction in a marine pelagic trench (Eloe et al., 2011) (**Figure 4** and Supplementary Figure S3).

Furthermore, the bacterial richness was significantly higher in the 1 μm fraction in December and November (**Figure 2**). This suggests that the diversity of the large/attached bacteria recovered from the 1 μm filter were greater in the later months (November and December) compared to the smaller/free-living bacterial communities of the 0.2 μm filter. This increased diversity in the larger fraction agrees with studies investigating the differences between the particle-associated and free-living bacteria in other aquatic systems, such as the Baltic and Mediterranean Seas (Crespo et al., 2013; Rieck et al., 2015). While the alpha diversity trends were similar between the two filter fractions, the increasing richness was more prominent in the 1 μm filter fraction (**Figure 2**). There can be several factors that may influence this discrepancy in temporal diversity, such as changing protozoan grazing rates and/or viral lysis impacting the microbial food web (Šimek et al., 1999; Hahn and Höfle, 2001; Weinbauer et al., 2007). Additionally, it can be suggested that the attached/large bacteria of the 1 μm fraction may have been better equipped to compete in an environment of decreased water temperature, nitrate, and chloride levels (**Figure 1**). In fact, previous studies have reported particle-associated bacteria have larger genomes compared to streamlined free-living bacteria (Smith et al., 2013). This reservoir of genetic material may allow for a more adaptive lifestyle in the face of changing conditions, competing bacterial populations and predators.

The viral fraction was dominated by sequences homologous to the tailed, double stranded DNA bacteriophage of the order *Caudovirales* (**Figure 5A**). Within the *Caudovirales* order, however, the family of generally temperate *Siphoviruses* was the most abundant at each time point (**Figure 5A**). This is in contrast to other freshwater systems, where *Myoviruses* and *Podoviruses* (Lakes Ontario, Erie, Matoaka, and Michigan (Fancello et al., 2013; Green et al., 2015; Mohiuddin and Schellhorn, 2015) were found to dominate. Conversely, *Siphoviruses* were reported to be abundant in sediments (Breitbart et al., 2004) and terrestrial subsurface environments (Edwards and Rohwer, 2005). *Siphovirus* abundance within the pond water sampled here may reflect the pond’s unique topology compared to larger lakes. Ponds and small lakes have a higher terrestrial-aquatic interchange than larger freshwater systems due to their close contact with the adjacent terrestrial environment (Palik et al., 2001; Søndergaard et al., 2005). This large littoral zone may promote the influx of terrestrial/sediment *Siphoviruses.* Additionally, because the dominant lifecycle of cultured *Siphoviruses* (Suttle, 2005) is lysogenic, their abundance may be indicative of environmental stress, (e.g., changes in nutrients, pH or temperature) activating the lytic-lysogenic switch and thus increasing their presence in the free phage fraction (McDaniel et al., 2002). This agrees with the phylogenetic analysis of the informative viral marker gene family A DNA polymerase, *polA* (Pol I protein). In this case, the Pol I proteins were temporally persistent and dominated by the Leu762 mutation, suggested to be indicative of temperate phage (**Figure 6**) (Schmidt et al., 2014).

This abundance of temperate phage is important to note as they can alter the genetic architecture of their hosts, which can in turn influence the surrounding microbial community and environment. For instance, temperate phage can transduce bacterial DNA and potentially alter host biology, such as maintaining host photosynthesis during infection or encoding virulence and antibiotic resistance genes (Wagner and Waldor, 2002; Lindell et al., 2004; Balcazar, 2014). In this case, while present, genes involved in virulence/antimicrobial resistance were not enriched within the viral fraction during the study period (**Figure 5B**). However, the samples did contain a high abundance of genes involved in Nucleosides & Nucleotides production, largely ribonucleotide reduction genes. This is not surprising as ribonucleotide reductases (RNRs) have been observed frequently within aquatic viral metagenomic libraries and namely in *Myoviruses* (Angly et al., 2006; Bench et al., 2007; Wommack et al., 2015).

From these data we were able to characterize the bacterial and viral taxonomic and functional components within an agricultural pond over time. In several cases, we observed the abundance of the dominant viral families correlated with the relative abundance of bacterial taxa. However, more work is necessary to track and model these interacting species. A more thorough analysis connecting phage with their hosts, such as through the use of the Clustered regularly interspaced short palindromic repeats (CRISPR) system, is warranted to provide a more nuanced assessment of their relationship in pond water and potential impact in the microbial food web (e.g., viral shunt). Additionally, other parameters like phosphate, dissolved organic carbon, chlorophyll concentration, and protozoan grazing rates might also exert some influence on the temporal dynamics observed here and should be included in future studies of freshwater ponds. Overall, this analysis serves as a baseline of the diversity and dynamics of three distinct microbial fractions in agricultural pond water.

## Author Contributions

JC performed processing of water samples following collection, conducted the majority of the bioinformatic and biostatistical analyses following sequence processing, and wrote and edited the manuscript. DN performed metagenomic assemble and abundance calculations. SA and AB participated in the sample collection and aided in DNA extraction. AS and EM contributed to the study design, protocol development, data analysis and interpretation, and manuscript preparation. All authors read, edited, and approved the final manuscript.

## Conflict of Interest Statement

The authors declare that the research was conducted in the absence of any commercial or financial relationships that could be construed as a potential conflict of interest.
