# Supplementary material for: Agricultural Freshwater Pond Supports Diverse and Dynamic Bacterial and Viral Populations
Source: Front Microbiol. 2018 Apr 24;9:792. doi: 10.3389/fmicb.2018.00792 (PMC5928236; doi:10.3389/fmicb.2018.00792)
Supplement: Supplementary file 1 [file Data_Sheet_1.DOCX]

**Table S1**: Difference (%) in relative abundance between 1 μm and 0.2 μm fractions for October, November, and December for the dominant bacterial phyla

| **Phlya** | **October** | **November** | **December** |
| --- | --- | --- | --- |
| *Proteobacteria* | 1.65* | 2.93** | 3.89** |
| *Actinobacteria* | -6.42** | -7.72** | -6.67** |
| *Bacteroidetes* | 0.19 | 1.24** | -1.67* |
| *TM7* | 0.75** | -0.44** | 0.26 |
| *Cyanobacteria* | 1.55** | 2.13** | 2.38** |
| Unknown | -0.53 | -0.24 | -0.82** |
| *OD1* | -0.7 | -0.46 | -1.91** |
| *Verrucomicrobia* | 0.23 | 0.42** | 1.09** |
| *Firmicutes* | 0.83** | 0.55** | 0.62** |
| *Chloroflexi* | 0.83** | 0.56** | 1.35** |
| Other | 1.6* | 1.02 | 1.49** |
| *p <0.05, **p <.01 | |  |  |

**Table S2**: Difference (%) in relative abundance of dominant bacterial phyla between sampling dates in 1 μm and 0.2 μm filter fractions

| **Phyla** | **1 μm** | | | **0.2 μm** | | |
| --- | --- | --- | --- | --- | --- | --- |
|  | **OCT-NOV** | **NOV-DEC** | **OCT-DEC** | **OCT-NOV** | **NOV-DEC** | **OCT-DEC** |
| *Proteobacteria* | 1.42 | -5.5** | -4.08** | 2.7* | -4.55** | -1.85 |
| *Actinobacteria* | -6.44** | 0.073** | 0.83 | -7.74** | 8.33** | 0.58 |
| *Bacteroidetes* | -1.28* | -0.05 | -1.33* | -0.22 | -2.96** | -3.19** |
| *TM7* | 0.95** | 0.37* | 1.32** | -0.24 | 1.07** | 0.83** |
| *Cyanobacteria* | 2.58** | -0.23 | 2.35** | 2.79** | -0.36 | 2.8** |
| Unknown | -0.31 | 0.34 | 0.02 | -0.03 | -0.24 | -0.27 |
| *OD1* | -1.03* | 1.33** | 0.29 | -0.79 | -0.13 | -0.92 |
| *Verrucomicrobia* | 0.85** | -0.98** | -0.13 | 1.03** | -0.31 | 0.72** |
| *Firmicutes* | 0.32 | -0.19 | 0.13 | 0.04 | -0.13 | -0.09 |
| *Chloroflexi* | 0.56** | -0.88** | -0.32* | 0.29** | -0.09 | 0.2* |
| Other | 2.39** | -1.47** | 0.92 | 1.81* | -1 | 0.8 |
| *p <0.05, **p <.01 | | |  |  |  |  |

**Table S3**: Difference (%) in relative abundance between 1 μm and 0.2 μm fraction for October, November, and December for the dominant bacterial genera.

| **Genera/Taxa** | **October** | **November** | **December** |
| --- | --- | --- | --- |
| *Actinomycetales; ACK.M1* | -5.74** | -3.96** | -5.31** |
| *Acidimicrobiales; C111* | -0.39 | -0.76** | -0.58** |
| *Limnohabitans* | -1.34** | -0.74** | -1.75** |
| *Microbacteriaceae* | -0.43** | -1.07** | -0.64** |
| *Synechococcus* | 1.35** | 0.74** | 0.35** |
| *Comamonadaceae* | -0.33 | 0.17 | -0.23 |
| *Fluviicola* | 0.01 | 0.22 | -0.62** |
| *Chitinophagaceae* | 1.24** | 0.24* | 0.40** |
| *Sediminibacterium* | -0.44** | -0.35** | -0.80** |
| *Polynucleobacter* | -0.99** | -0.42** | -0.69** |
| *Sphingomonadales* | 0.00002 | -0.09 | -0.66** |
| *Actinomycetales*; other | -0.69* | -0.55** | -0.73** |
| *Rhodoluna* | -0.29 | -0.59** | -0.28** |
| *Sphingobacteriales* | -0.3* | 0.21 | -0.42** |
| *Dolichospermum* | 0.2** | 1.16** | 1.67** |
| *Flavobacterium* | -0.02 | 0.11 | -0.23** |
| *Cytophagaceae* | -0.36** | -0.18* | -0.57** |
| *Actinomycetales* | 0.28 | -0.27** | 0.03 |
| *TM7; TM7.1* | 0.41* | -0.17 | 0.28 |
| *Rhodobacter* | 0.03 | 0.47** | 0.95** |
| *OD1; ZB2* | -0.4** | -0.27* | -0.76* |
| *Rhizobiales* | 0.3** | 0.15* | 0.55** |
| *Mycobacterium* | 0.28* | -0.28** | 0.02 |
| *Rhizobiales; other* | 0.06 | 0.06 | 0.23** |
| *Bacteroidetes* | -0.25 | 0.02 | -0.28** |
| *TM7; SC3* | 0.21** | -0.18** | 0.05 |
| *Betaproteobacteria; SC.I.84* | 0.63** | 0.42** | 1.24** |
| *OD1; ABY1* | -0.16 | -0.17 | -0.61** |
| *Betaproteobacteria* | -0.2* | -0.09 | -0.39** |
| *Gemmataceae* | 0.33** | 0.13** | 0.2** |
| *OD1* | 0.04 | -0.03 | -0.38** |
| *p <0.05, **p <.01 | |  |  |

**Table S4**: Difference (%) in relative abundance of dominant bacterial genera between sampling dates in 1 μm and 0.2 μm filter fractions

| **Taxa** | **1 μm fraction** | | | **2μm fraction** | | |
| --- | --- | --- | --- | --- | --- | --- |
|  | **OCT-NOV** | **NOV-DEC** | **OCT-DEC** | **OCT-NOV** | **NOV-DEC** | **OCT-DEC** |
| *Actinomycetales; ACK.M1* | -9.17** | 4.21** | -4.96** | -7.39** | 2.86** | -4.53** |
| *Acidimicrobiales; C111* | 0.54** | 0.99** | 1.53** | 0.17 | 1.17** | 1.34** |
| *Limnohabitans* | -0.46** | 0.73** | 0.27 | 0.14 | -0.28 | -0.13 |
| *Microbacteriaceae* | -0.77** | 0.52** | -0.24 | -1.4** | 0.95** | -0.45 |
| *Synechococcus* | 3.34** | 0.61** | 3.94** | 2.73** | 0.22* | 2.95** |
| *Comamonadaceae* | -0.52** | -0.08 | -0.6** | -0.02 | -0.48 | -0.5 |
| *Fluviicola* | -1.7** | 1.23** | -0.48** | -1.5** | 0.39* | -1.11** |
| *Chitinophagaceae* | 1.48** | -0.3** | 1.18** | 0.48** | -0.14 | 0.34* |
| *Sediminibacterium* | -0.68** | 0.43* | -0.25 | -0.59** | -0.02 | -0.61** |
| *Polynucleobacter* | -0.19** | -0.3** | -0.49** | 0.38** | -0.58** | -0.19 |
| *Sphingomonadales* | -1.19** | 0.2** | -0.99** | -1.28** | -0.38** | -1.65** |
| *Actinomycetales*; other | -0.51** | 0.65** | 0.14 | -0.38 | 0.48 | 0.1 |
| *Rhodoluna* | 0.41* | 0.85** | 1.26** | 0.11 | 1.16** | 1.28** |
| *Sphingobacteriales* | 0.41** | -0.14 | 0.27* | 0.92** | -0.77** | 0.15 |
| *Dolichospermum* | -0.93** | -0.67** | -1.6** | 0.04 | -0.16** | -0.12* |
| *Flavobacterium* | 0.39* | -0.69** | -0.3* | 0.53** | -1.03** | -0.5** |
| *Cytophagaceae* | -0.46** | 0.43** | -0.04 | -0.29** | 0.03 | -0.25** |
| *Actinomycetales* | 0.59** | -0.09 | 0.51** | 0.04 | 0.22 | 0.26* |
| *TM7; TM7.1* | 0.08 | -0.14 | -0.07 | -0.51** | 0.3 | -0.2 |
| *Rhodobacter* | 0.66** | -0.37** | 0.28** | 1.1** | 0.1 | 1.2** |
| *OD1; ZB2* | -0.62** | .40** | -0.22 | -0.49 | -0.09 | -0.58* |
| *Rhizobiales* | 0.89** | -0.08 | 0.81** | 0.74** | 0.32** | 1.06** |
| *Mycobacterium* | 0.93** | 0.34** | 1.26** | 0.44** | 0.64** | 1.08** |
| *Rhizobiales;* other | 0.51** | 0.02 | 0.52** | 0.51** | 0.19* | 0.7** |
| *Bacteroidetes* | -0.79** | 0.26 | -0.53** | -0.52** | -0.03 | -0.55** |
| *TM7; SC3* | 0.76** | 0.36** | 1.12** | 0.37** | 0.59** | 0.95** |
| *Betaproteobacteria; SC.I.84* | 0.19* | -0.89** | -0.7** | -0.01 | -0.07* | -0.08** |
| *OD1; ABY1* | -0.28 | 0.35 | 0.07 | -0.29 | -0.09 | -0.38* |
| *Betaproteobacteria* | -0.33** | 0.11 | -0.23** | -0.23** | -0.19* | -0.42** |
| *Gemmataceae* | 0.67** | 0.1* | 0.76** | 0.47** | 0.16** | 0.63** |
| *OD1* | 0.02 | 0.39* | 0.41* | -0.04 | 0.03 | -0.01 |

*p <0.05, **p <.01


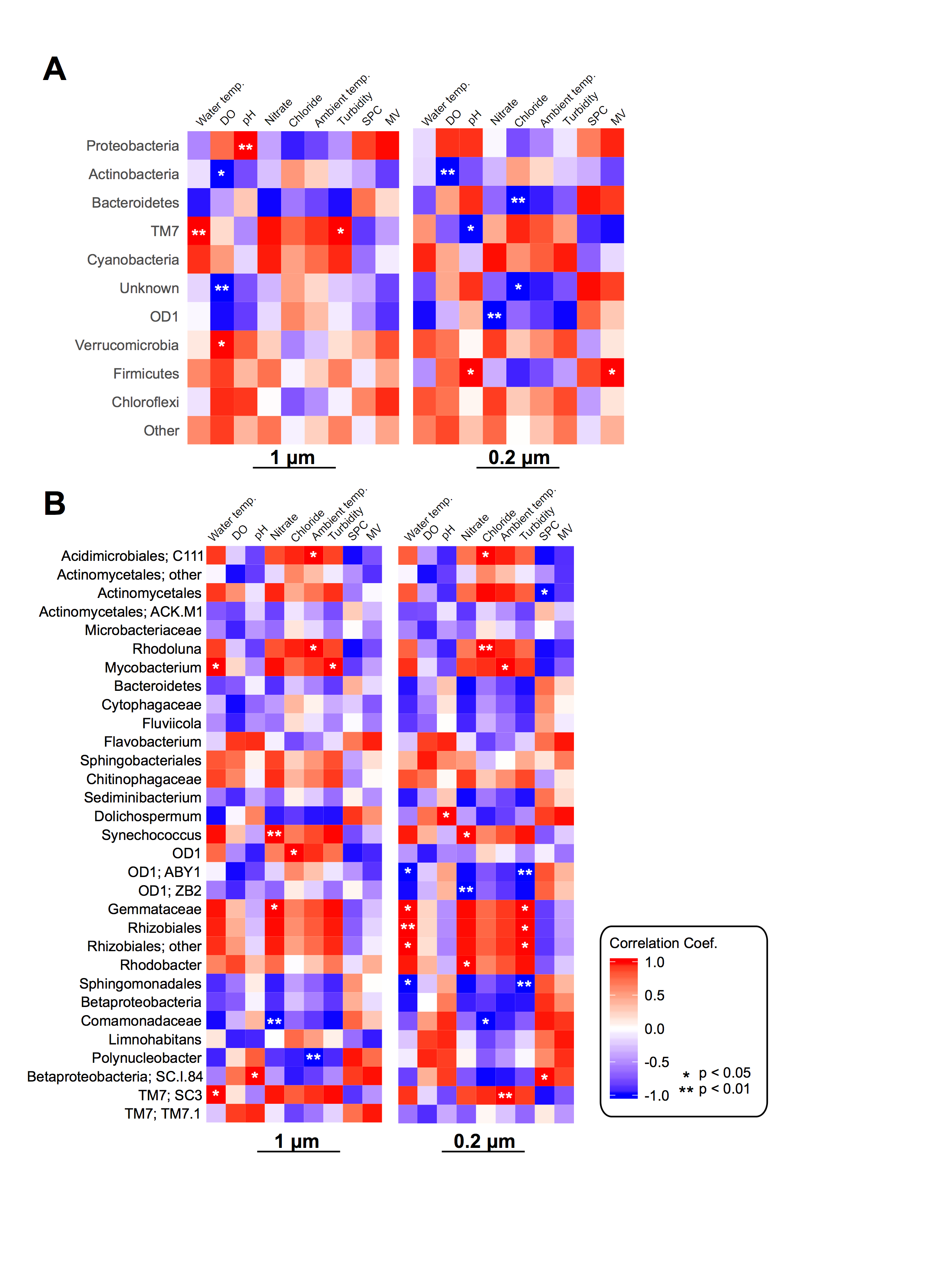


**Figure S1**: Heatmaps of the Pearson’s correlation coefficients between the water characteristics and relative abundance of bacterial (A) phyla and (B) genera for the 1 μm and 0.2 μm filter fractions. Color gradients reflect the different values of Pearson’s correlation coefficients. MV: Oxidation/reduction (mV), SPC: Conductivity (SPC uS/cm), DO: Dissolved Oxygen (%)


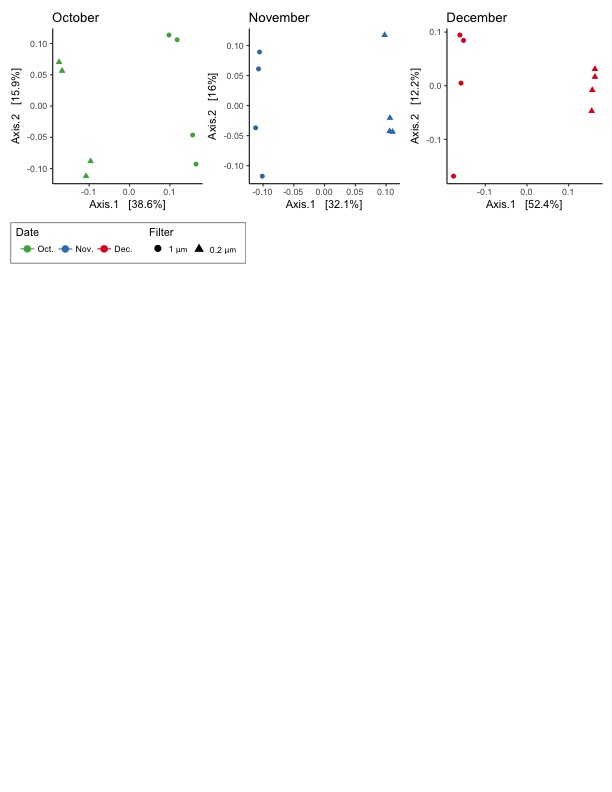


**Figure S2:** PCoA plots of beta diversity (by date) measured using Bray-Curtis. Shape denotes filter pore size, either 1μm (circle) or 0.2μm (triangle), and color denotes the month that water was sampled, October (green), November (blue), and December (red).

**Figure S3:** Core *Actinobacteria*, *Proteobacteria*, and *Bacteroidetes* OTUs for 1 μm and 0.2 μm filter fractions during the entire sampling period. Krona plots depicting the core OTUs in the (A) 1μm and (B) 0.2μm filter fractions for those assigned to the *Actinobacteria*, *Proteobacteria*, and *Bacteroidetes* phyla*.*

*
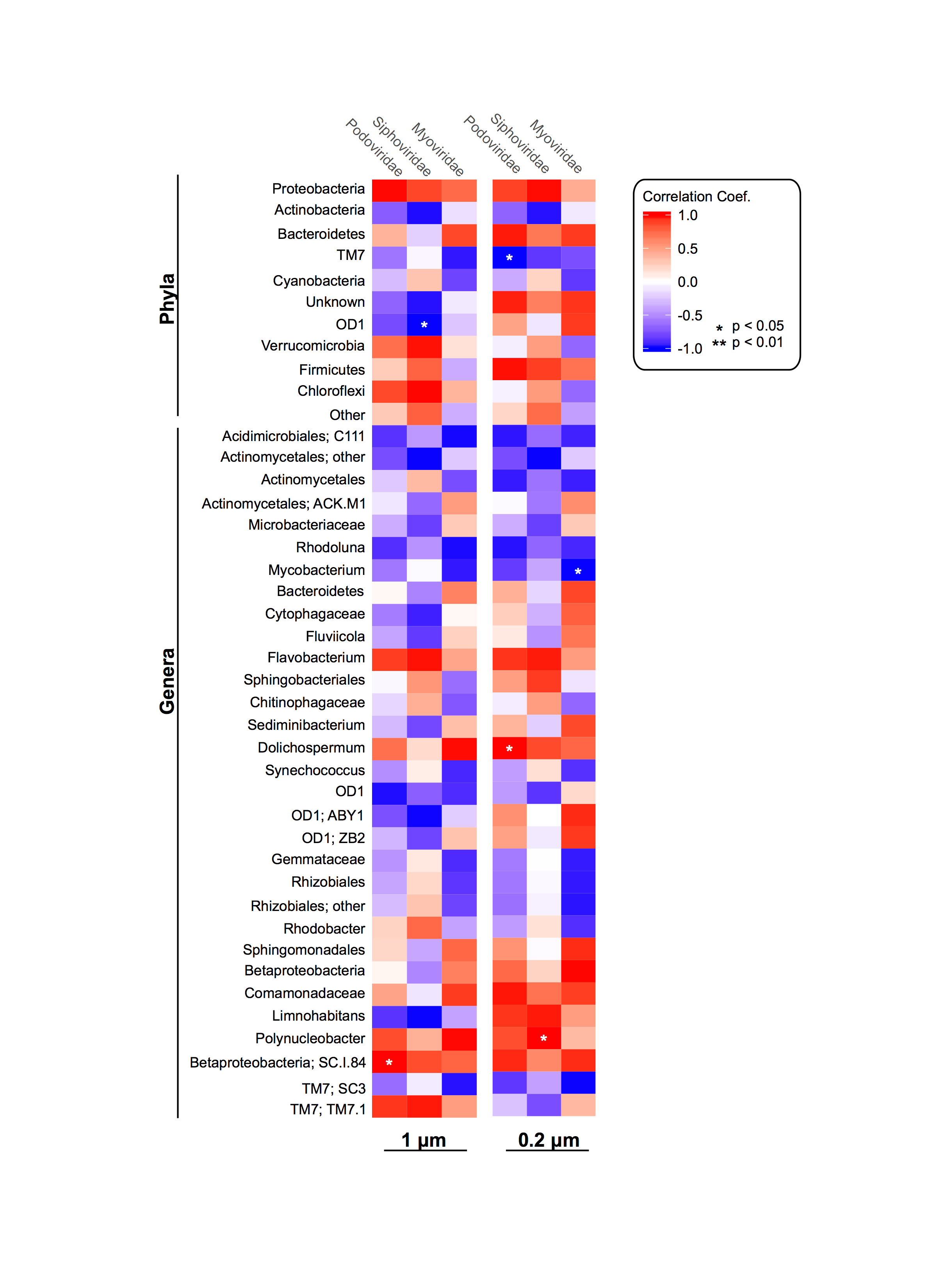
*

**Figure S4**: Heatmaps of the Pearson’s correlation coefficients between dominant viral families and the relative abundance of bacterial phyla and genera in both the 1 μm and 0.2 μm filter fractions. Color gradients reflect the different values of Pearson’s correlation coefficients.
